# Advanced Membrane Technologies and Hybrid Treatment Systems for Sustainable Removal of Naturally Occurring Radioactive Materials from Industrial Wastewater

**DOI:** 10.3390/membranes16040125

**Published:** 2026-03-31

**Authors:** Amal S. Al Saadi, Ismail Al-Yahmadi, Sharif H. Zein, Natarajan Rajamohan, Intisar K. Al-Busaidi, Nabila Al-Rashdi, Safa Al Habsi, Saada Al Shukaili, Ali Alawi, Rashid Al Mashrafi

**Affiliations:** 1 Chemical Engineering Section, Faculty of Engineering, Sohar University, Sohar 311, Oman; s.h.zein@hull.ac.uk (S.H.Z.); rnatarajan@su.edu.om (N.R.); 2 Department of Physics, Sultan Qaboos University, Muscat 123, Oman; 3 School of Engineering, Chemical Engineering, Faculty of Science and Engineering, University of Hull, Kingston Upon Hull HU6 7RX, UK; 4 Oil and Gas Section, University of Technology and Applied Science, Muscat 133, Oman; intisar.albusaidi@utas.edu.om; 5 Chemical Engineering Section, National University of Science & Technology, Muscat 111, Oman; nabilaalrashdi@nu.edu.om; 6 Water Management, Petroleum Development Oman LLC, Muscat 100, Oman; 7 Corporate Environment, Petroleum Development Oman LLC, Muscat 100, Oman; 8 Occupational Health, Petroleum Development Oman LLC, Muscat 100, Oman

**Keywords:** NORMs, radioactive wastewater, membrane filtration, radium separation, electrocoagulation, sustainable remediation

## Abstract

Naturally Occurring Radioactive Materials (NORMs) in industrial wastewater present significant environmental and public health challenges due to their persistence and radiotoxic effects. This comprehensive review analyzes 108 peer-reviewed publications from 2014 to 2025 on NORM treatment technologies for industrial wastewater. While previous reviews have focused on individual treatment methods or laboratory-scale studies, this work provides comparative performance analysis across multiple technologies under realistic industrial conditions, including high-salinity environments and competing ions. We emphasize membrane filtration, electrocoagulation (EC), ion exchange, and advanced oxidation processes, evaluating both their economic feasibility and environmental sustainability for practical industrial implementation. The review discusses the advantages and limitations of existing techniques, highlighting the need for integrated strategies that combine physical, chemical, and biological processes for enhanced remediation. Hybrid systems combining multiple technologies outperform individual approaches by 15–25% in removal efficiency. These advances are critical for ensuring safe water reuse and protecting water resources from radioactive contamination. Additionally, regulatory frameworks governing NORM management are examined, underscoring the importance of standardized disposal and treatment protocols. The review concludes by identifying research gaps and future directions. Priority areas include developing standardized treatment protocols and strengthening academia–industry collaboration to achieve scalable solutions aligned with UN Sustainable Development Goal 6.

## 1. Introduction to NORMs

The worldwide management of radioactive contaminants in industrial wastewater has become a major environmental issue because it threatens the health of millions who live in oil, gas, and mining areas. NORMs exist in the Earth’s crust, mainly as low-abundance levels of uranium and thorium minerals distributed through geological layers. These materials exist in nature at normal levels, but human activities cause their concentration to rise, which creates dangerous situations when they enter water systems. These NORMs exist within their original sources as naturally occurring radioactive elements until industrial processes elevate their concentrations into Technologically Enhanced Naturally Occurring Radioactive Materials (TENORMs) through mining operations and oil extraction pursuits, as well as industrial manufacturing activities [1,2]. The extraction of oil and gas through industrial processes leads to dangerous radiological risks because these operations produce and accumulate these materials, which threaten both personnel operating in the area and people living nearby [1,3]. Research indicates that extraction sites have already started to show environmental impacts in surrounding ecosystems. Scientists have documented elevated uranium and thorium isotopes in fish populations from the Athabasca Oil Sands Region, which leads to observable growth and reproductive problems [4]. Health and environmental risks from NORMs and TENORMs demand proper management alongside proper monitoring for risk control [2,5]. The documented risks of NORM contamination in industrial wastewater streams have not led to sufficient treatment methods that can handle the extensive nature of this problem.

Radiologic substances from naturally occurring radioactive materials (NORMs) include uranium, thorium, and radium found in the planet’s surface. NORMs exist primarily in mining waste products alongside emissions generated by domestic gas activities and geological rock formations. The materials that occur naturally in the Earth’s crust tend to accumulate during mineral extraction, during the production of oil and gas, and during water treatment operations. The discharge of NORM-contaminated materials by either wastewater systems or industrial waste streams creates major environmental threats and potential harm to public health [6]. The current treatment approaches fail to effectively handle these intricate contamination situations. Conventional methods such as chemical precipitation and basic filtration are hindered by high salinity (total dissolved solids (TDS) > 30,000 mg/L) common in oil- and gas-produced water, competing ions (Ca^2+^, Mg^2+^) that reduce selectivity, and the formation of secondary radioactive waste requiring specialized disposal. These limitations necessitate advanced treatment technologies capable of operating under challenging industrial conditions.

### 1.1. Significance for Environmental and Public Health

NORMs in wastewater present a major risk because they have the potential to cause both radiological injuries to human populations and environmental damage. Radionuclides in NORMs produce alpha, beta, or gamma radiation that creates health dangers for people who ingest or inhale them or come into contact through skin exposure. Wastewater containing NORM pollutants accumulates within aquatic organisms, thus damaging ecosystem stability before finding its way into food web pathways. Reference levels that remain elevated for sustained periods boost the chances of cancer appearance and organ damage, along with genetic anomalies in human beings and wildlife populations [7].

### 1.2. Regulations Related to NORM Exposure

Multiple countries have established governing laws to control NORM waste management and exposure safeguards. The United States Environmental Protection Agency (EPA), together with the International Atomic Energy Agency (IAEA), creates frameworks that outline both threshold exposure amounts and recommended waste disposal methods. Manufacturers must measure NORM concentrations within their facilities while building treatment systems for radioactive wastewater and following approved waste manipulation and storage guidelines [8]. Technical resolutions that eliminate NORM contaminants are vital to protecting both the environment and human health because they prevent industrial operations from causing ecological degradation or harmful exposure.

### 1.3. Needs for Advanced Treatment Technologies

The necessity for efficient ways to manage NORMs in wastewater becomes critical as environmental and health threats from pollutants, along with new contaminants and micropollutants, surge. Growing industrial activities and urban expansion cause untreated wastewater discharges to create major ecological problems and health dangers, which demand advanced treatment systems to control these destructive outcomes [9]. The persistent character of these contaminants expands beyond the capabilities of standard treatment methods, which allow the wastes to build up in natural systems and generate ongoing health risks for people, according to Saleem et al. [10]. Researchers Ahmad et al. [11] and Zafar and Aqeel [12] describe bioremediation with phytoremediation alongside AOPs as necessary technologies for pollutant degradation to meet environmental safety standards. The application of sustainable methodologies in wastewater processing matches global environmental objectives while developing a circular economy that converts waste into reusable materials [9,10]. The creation of powerful treatment methods remains essential to protect both public health standards and maintain environmental systems. Membrane technology and sustainable treatment methods have caused recent progress, which indicates they can solve these issues [13]. This review tackles these problems through an evaluation of present and future treatment solutions.

### 1.4. Regulatory Framework for NORM Management

Regulatory limits for NORM discharge vary considerably across jurisdictions, complicating technology selection and implementation. The US EPA establishes uranium limits at 30 μg/L for drinking water, while industrial discharge limits typically range from 100 to 1000 μg/L depending on the receiving water body. Combined radium-226 and radium-228 limits are set at 5 pCi/L (0.185 Bq/L) for drinking water. The IAEA provides international guidance, though individual nations maintain their own enforceable standards.

Technology selection must consider three regulatory dimensions: whether treated effluent meets discharge standards, classification and disposal requirements for radioactive waste streams (concentrated brine, spent adsorbents, contaminated sludge), and ongoing monitoring obligations. Technologies achieving >95% removal may still fail regulatory compliance if feed concentrations are exceptionally high, necessitating multi-stage treatment. Furthermore, the radioactive waste generated during treatment often presents greater disposal challenges and costs than the treatment process itself, particularly for radium-contaminated materials requiring specialized handling and licensed disposal facilities.

### 1.5. Review Methodology

This review systematically examines NORM treatment technologies through structured literature analysis. We searched the Web of Science, Scopus, and Google Scholar from January 2014 to December 2025 using keywords “NORM removal,” “radioactive wastewater,” “uranium removal,” “radium removal,” and “thorium removal,” combined with technology terms such as “membrane,” “adsorption,” “electrocoagulation,” and “ion exchange.”

Studies were included if they reported quantitative removal efficiencies for uranium, radium, or thorium with specified operating conditions (pH, temperature, contact time, or salinity). We prioritized research using industrial wastewater or representative synthetic solutions, and excluded purely theoretical studies, bench-scale experiments without scale-up discussion, or those lacking methodological detail. From each study, we extracted technology type, removal efficiency, operating parameters, feed water characteristics, operational scale, and energy consumption where available. A total of 108 peer-reviewed publications met these criteria and form the basis of this review.

### 1.6. Review Objectives and Novelty

This review provides a comprehensive comparison of NORM removal technologies with quantitative performance data from industrial applications. Unlike previous reviews focusing on single technologies or laboratory studies, this work evaluates both technical performance and economic–environmental sustainability across multiple treatment approaches. We particularly emphasize challenging high-salinity conditions (TDS > 30,000 mg/L) common in oil and gas operations and identify critical technology gaps to guide future research and practical implementation.

Several recent reviews have addressed radioactive wastewater treatment from different perspectives. Ma et al. [14] provided a broad overview of treatment technologies but focused primarily on nuclear facility effluents. Alsarayreh et al. [15] examined membrane processes for liquid radioactive wastes, while Zhuang and Wang [16] concentrated specifically on cesium removal. Sharma et al. (2024) [17] reviewed radioactive elements in wastewater with emphasis on detection methods, and Alhamd et al. [18] addressed NORMs in Iraqi oil and gas operations. However, these reviews either focused on nuclear wastewater rather than NORMs from industrial sources, examined single technologies without comparative analysis, or lacked quantitative performance data under industrial conditions. This review distinctively compares multiple technologies under realistic industrial scenarios, particularly high-salinity environments (TDS > 30,000 mg/L) common in oil and gas operations and integrates technical performance with economic and environmental considerations to guide practical implementation.

## 2. Current Technologies for NORM Removal

Throughout this review, selectivity refers to a technology’s preferential removal of target radionuclides over competing ions. Selectivity is quantified using the selectivity coefficient (K_sel), defined as the ratio of target radionuclide uptake to competing ion uptake at equilibrium: K_sel = (q_target/C_target)/(q_competing/C_competing), where q represents the adsorbed amount and C represents equilibrium concentration. For membrane processes, selectivity is expressed as the ratio of rejection coefficients. Higher selectivity values indicate better discrimination between radionuclides and competing ions such as Ca^2+^, Mg^2+^, and SO_4_^2−^. A collection of advanced treatment approaches exists to eliminate NORMs from wastewater, including EC and AOPs, along with physical and biological solutions. The current research shows that hybrid treatment systems, which combine multiple methods, produce better performance results [19]. EC stands out in NORM removal by coprecipitating uranium and radium with aluminum/iron hydroxides while producing less radioactive sludge than standard chemical coagulation methods and demonstrating the ability to adapt to different wastewater types using operational controls for pH alongside current density [20,21]. Similarly, combining AOPs with ion exchange filtration systems delivers superior removal of organic-complexed radionuclides and uranium and radium [22]. Microorganisms such as algae and bacteria show potential for biosorption of uranium and radium, though their application for radioactive wastewater remains limited due to radioactive biomass disposal challenges. The combination of advancing methods targets wastewater quality improvements for reuse applications with decreased negative environmental consequences [22,23]. EC removes uranium (85–92%) and radium (78–85%) through coprecipitation at pH 6–8 and current density of 15–25 A/m^2^. Performance decreases significantly at high salinity (TDS > 50,000 mg/L) due to competing ions.

### 2.1. Different Technological Groups

Technologies for environmental remediation can be classified into three primary groups: physical, chemical, and biological methods. The use of physical methods, including adsorption together with air circulation and advanced oxidation processes, successfully removes radionuclides such as uranium and radium through adsorption and AOPs [24,25]. Chemical methods such as photocatalytic oxidation effectively degrade organic-complexed radionuclides, though careful control is needed to minimize toxic by-products [25,26]. Enzymes, including lactonase, together with microbial degradation and phytoremediation, show potential as sustainable methods for biosorption of radionuclides, though disposal of radioactive biomass remains a challenge [24,26]. These methods improve system effectiveness while demonstrating the value of combined strategies for environmental problem-solving [25]. Multiple studies confirm that integrated approaches combining physical, chemical, and biological methods achieve superior NORM removal compared to single-technology systems [27,28]. Ion exchange resins achieve uranium removal of 88–95% at pH 4–6. Performance reduces by 20–30% when competing ions (Ca^2+^, Mg^2+^) exceed 500 mg/L. Resins require regeneration after 10–15 cycles.

### 2.2. Advancements in NORM Removal Technologies

Reverse osmosis (RO), nanofiltration (NF), and ultrafiltration (UF) membrane technologies demonstrate high radionuclide removal efficiency. Functionalized membranes that integrate nanoscale substances such as graphene oxide or titanium dioxide show improved radionuclide filtration efficiencies together with boosted resistance to fouling [29,30]. Hybrid configurations combining membranes with ion exchange or AOPs achieve enhanced performance for eliminating uranium, radium, and thorium from complex NORM-contaminated waste streams [31]. Recent membrane innovations demonstrate selectivity for specific radionuclides, for extracting cesium and strontium from nuclear wastewater, as well as uranium and radium from oil- and gas-produced water [32,33,34]. RO achieves 94–97% NORM removal but requires high energy (4–6 kWh/m^3^). NF achieves 88–94% removal with lower energy (2–3 kWh/m^3^). Both suffer fouling at high organic content (TOC > 20 mg/L). Further details on membrane advantages, fouling challenges, and advanced nanotechnology variants are provided in Section 3 and Section 4. Membrane rejection of radionuclides occurs through size exclusion, electrostatic repulsion between charged membrane surfaces and ionic species, and complexation with functionalized groups [29]. Fouling from inorganic scaling (CaCO_3_, CaSO_4_, radium-containing precipitates), organic matter, and biofilms reduces flux over time, requiring periodic cleaning [35,36]. Radiation exposure can degrade polymer membranes through chain scission, though advanced materials show improved radiation resistance.

Sorbent materials, including zeolites, MOFs, and functionalized biochar, demonstrate high selectivity for radionuclide uptake across varying concentration conditions. While MOFs offer regeneration capabilities for uranium and radium capture [37], biochar and activated carbon provide lower-cost alternatives compared to MOFs, which achieve higher removal efficiency but at significantly greater expense.

**Figure 1:**
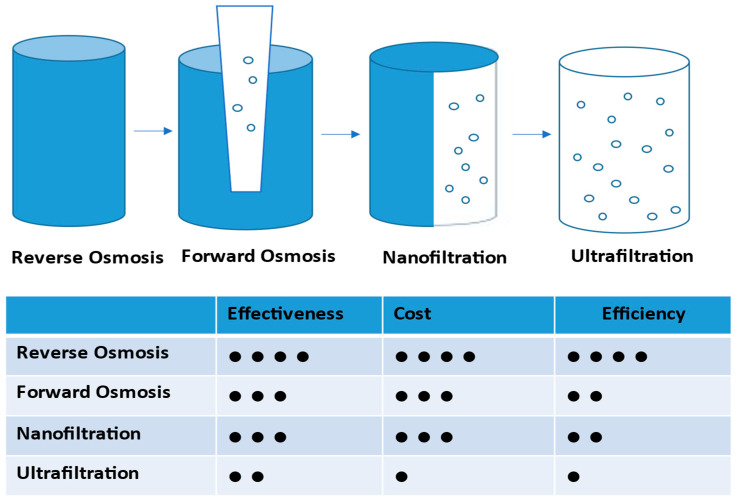
NORM removal technologies as a comparison of effectiveness, efficiency, and cost. Dots present the pore size of membranes.

AOPs such as photocatalysis and ozonation effectively degrade organic-complexed radionuclides. When integrated with membrane or sorption systems, AOPs create synergies that enhance overall performance. These hybrid approaches show particular promise for oil and gas wastewater with high salinity and organic content, where AOPs reduce membrane fouling by 40–60% and improve NORM removal by 10–15% [38]. Table 1 presents a comparative overview of removal efficiencies, operating conditions, and energy requirements for major NORM treatment technologies. Economic considerations play an important role in technology selection. Although Table 1 outlines the different treatment processes, published studies suggest that adsorption methods tend to have lower upfront costs but require frequent regeneration, while membrane technologies involve a higher initial investment with lower operating costs over time. The high material and disposal costs associated with advanced membranes often make hybrid systems a more practical option. Table 1 highlights key selection criteria. RO and NF achieve the highest removal efficiencies but consume more energy, while ion exchange and EC balance performance with moderate energy demands. Biochar and activated carbon offer economical alternatives despite lower removal rates. The TDS limits are particularly important, showing salinity thresholds where performance degrades. For high-salinity wastewater (TDS > 30,000 mg/L) common in oil and gas operations, forward osmosis (FO) and adsorption become more practical despite lower absolute removal efficiencies. Figure 1 compares NORM removal technologies across three key dimensions: treatment effectiveness, removal efficiency, and cost. This enables the selection of technologies that best balance performance and economics for specific applications.

## 3. Advantages and Disadvantages of Current Technologies

A critical evaluation of each technology’s advantages and limitations is essential for selecting appropriate treatment approaches based on wastewater characteristics and operational constraints. Membrane filtration offers high radionuclide removal efficiency across diverse wastewater types. Recent advances in functionalized membranes have improved fouling resistance and selectivity. Membrane filtration offers high radionuclide removal efficiency (88–97%) across diverse wastewater types [40]. However, fouling and scaling remain persistent challenges requiring frequent maintenance that reduces operational efficiency. Moreover, the use of high-energy pressure-driven systems, such as RO and NF, enhances the operational costs [41]. Additionally, concentrated radioactive brine requires specialized disposal, posing environmental and economic challenges [42]. Table 2 provides a quantitative comparison of key performance parameters and limitations to facilitate technology selection.

Nuclear waste remediation: A highly selective and low-cost sorbent for the targeted removal of specific radionuclides can be achieved with MOFs and biochar that can also be regenerated. These engineered sorbents can be developed based on specific applications to target NORM-contaminated waste streams and provide flexibility in the treatment processes [39]. Nevertheless, the comparative advantages of sorbent materials include low costs, ease of use, and the ability to combat hydrocarbon pollution of water on- and offshore. However, the adsorption capacity of sorbent materials diminishes in high salinity conditions. Furthermore, there is concern about how to dispose of used sorbents since they are environmentally sensitive. Additionally, there is the real possibility of desorption of the captured radionuclides, thus compromising the safety and efficiency of the treatment process [17].

Membrane fouling is quantified by flux decline over time. Studies show flux decreases by 15–25% over 30 days with proper pre-treatment, but can decline by 40–60% within 72 h without pre-treatment [35]. High organic content (TOC > 20 mg/L) accelerates fouling, requiring cleaning every 10–15 days compared to 30–45 days for low-TOC wastewater. Chemical cleaning using alkaline (pH 11–12) and acid (pH 2–3) solutions restores 85–95% of the initial flux, though recovery decreases after repeated cycles [36].

### 3.1. Criteria for Evaluating NORM Removal Technologies

Technology evaluation is based on four quantitative criteria: removal efficiency (target > 90%), cost-effectiveness (operating cost < $2/m^3^), environmental sustainability (energy consumption, waste generation), and operational simplicity (maintenance requirements, operator skill level. Efficiency stands as an essential performance measurement because it includes the speed at which radionuclides are removed from the water alongside its capability to deal with dissimilar wastewater compositions. The high efficiency of membrane technologies becomes limited when fouling occurs. Sorbents demonstrate exceptional selectivity, whereas AOPs degrade complex organics [43].

Evaluation of cost-effectiveness occurs through examination of capital expenses, operational expenses, and maintenance requirements. Sorbents and ion-exchange resins work most cost-effectively in smaller systems, yet membranes and combination systems function best for bigger industrial applications [44].

Sustainability evaluation focuses on environmental tech effects, together with waste disposal and resource consumption. Waste management practice improves with membranes built for low energy usage and reusable sorbents. The combination of AOPs with renewable energy systems brings achievable sustainability possibilities despite their elevated energy needs [45].

Ease of operation is the functionality assessment that focuses on both installation simplicity and device service and usage activities. The co-precipitation and ion exchange methods operate more simply than membrane systems and AOPs, which demand versatile operators with specialist monitoring equipment [46].

### 3.2. Membrane Materials: Bridging Gaps by Resolving Complex Waste Treatment Situations

Membrane-based solutions present significant possibilities for improving NORM removal from existing technological options. Novel MOFs, as well as graphene oxide and functionalized polymers, now enable researchers to design systems that promote both higher efficiency rates and sustainability levels [47]. Continuous research aimed at membrane system advancements and energy-efficient methods continues to be vital for improving traditional membrane technologies’ limitations. MOFs and COFs, microporous frameworks, thin-film membrane designs, and advanced polymeric composites have made significant progress in solving fouling resistance and separation efficiency problems [48,49,50]. Membranes, when used in combination with technologies like AOPs or ion exchange, create hybrid solutions that help tackle complex NORM contamination in NORM removal. Research on innovative removal methods advances radionuclide extraction while creating economical, sustainable solutions for industrial radioactive waste management [51].

## 4. Nanotechnology Membranes

Due to their nanomaterial design, nanotechnology membranes deliver groundbreaking results in filtration and separation improvement because these membranes exhibit superior performance over conventional ones. The membranes possess engineered characteristics that provide great surface area and superior permeability, along with selectivity. The primary factor behind a nanotechnology membrane’s function lies in nanomaterials such as carbon nanotubes (CNTs), graphene oxide, and MOFs, together with nanoparticles, since these elements deliver exceptional molecular-level filtration control [52].

Nanotechnology membrane designs require the combination of nanoscale substances with either polymer or ceramic frameworks. Advanced materials undergo specific functional processes that enable them to detect and isolate particular harmful substances, including uranium, radium, and other radionuclides. Graphene oxide membranes utilize two-dimensional structures with atomic-scale design elements, which enable precise removal of ions while streamlining water flow through them. CNT-based membranes use their tube-like structure to enable fast transfer of water particles, which operates with less energy input compared to classical RO membranes (Wang et al., 2020) [53]. Under UV light exposure, titanium dioxide nanoparticles emerge as self-cleaning nanomaterials since they activate organic decomposition. Nanotechnology membranes deliver superior filtration capacities and exhibit outstanding chemical endurance and thermal stability, which positions them for optimal performance in demanding applications such as industrial NORM removal and brine management [54]. The combination of high efficiency and strong durability makes these materials viable options to tackle worldwide clean water needs.

Research conducted in 2025 demonstrates that graphene oxide membrane structures have evolved through ionic liquid insertion to enhance desalination performance [55]; carbon nanoparticle composite structures improve water purification [28]; and multifunctional Janus membranes perform both filtration and energy generation operations [56]. The NF process reaches exceptional performance levels when covalent organic frameworks (COFs) are integrated with reduced graphene oxide [57] and biopolymeric membranes containing carbon nanomaterials serve as environmentally friendly water treatment solutions [58].

### Applications of Nanotech Membranes in NORM Removal

Nanotechnology membranes improve NORM removal through high-efficiency separation that reduces membrane fouling, along with decreased power requirements for operation. Through different processing paths, including desalination and heavy metal removal, nanomaterials achieve enhanced sustainability and pollution degradation.

Graphene Oxide Membranes: Graphene oxide membranes present superior performance for the segregation of both radionuclides and salts from water. A pilot study revealed that graphene oxide membranes achieved salt rejection levels above 95% when desalinating brackish water alongside high-water flux measurements [59]. The ultrathin design of these materials decreases energy consumption while their biofouling resistance lowers maintenance expenses to enhance overall operational performance.

Carbon Nanotube (CNT) Membranes: CNT membranes function with nanoscale tubular structures to ensure outstanding water movement capacities. These materials demonstrate high efficacy in eliminating heavy metals such as lead, mercury, and cadmium. The 99% metal removal effectiveness was confirmed for electronics wastewater through electrofiltered CNT membranes that maintain high efficiency with minimal energy consumption [60].

MOFs: MOF membranes successfully eliminate both organic and inorganic substances from solutions. Textile dye NORM removal in China reached more than 90% pollutant removal through MOFs during pilot testing [61]. The membranes show robust performance with temperature and pH fluctuations, which extends their life span in difficult conditions.

Titanium Dioxide (TiO_2_) Nanoparticles: Photocatalysis allows TiO_2_-enhanced membranes to destroy NORMs while maintaining clean surface conditions. European municipal wastewater plants increased their ability to eliminate pharmaceuticals and endocrine-disrupting chemicals. UV photo-regeneration helps extend membrane lifespans while simultaneously boosting their operational effectiveness [62].

The novel nanotech-based filtration membranes demonstrate considerable promise for sustainable, efficient wastewater management across multiple practical uses.

Comparative performance analysis shows graphene oxide membranes achieve uranium removal of 95–97% at energy consumption of 3.5–4.5 kWh/m^3^, while CNT-based membranes achieve 92–95% removal at 2.8–3.8 kWh/m^3^. MOF-incorporated membranes demonstrate high selectivity (90–96% removal) but face cost challenges ($500–2000/kg material). TiO_2_ photocatalytic membranes provide self-cleaning capability, reducing fouling by 40–50% under UV exposure, though their uranium removal efficiency (85–90%) is lower than that of graphene oxide membranes [35,36].

Critical comparison reveals key trade-offs among nanomembrane materials. Graphene oxide membranes achieve the highest removal efficiencies but face manufacturing scalability and cost challenges. CNT membranes offer reduced energy consumption yet encounter difficulties with industrial-scale production and consistent dispersion. MOF-incorporated membranes demonstrate exceptional selectivity but require further validation for long-term stability under high-salinity industrial conditions. A notable gap across all nanotechnology membranes is the scarcity of long-term performance data beyond 6 months of continuous operation, limiting confidence for commercial deployment.

## 5. Forward Osmosis (FO)

FO merits separate discussion as it operates via osmotic gradient rather than hydraulic pressure, offering distinct advantages for high-salinity NORM wastewater (TDS > 30,000 mg/L) where energy consumption of pressure-driven membranes becomes prohibitive. The emerging membrane-based FO technology employs osmotic pressure gradients to transport water across semi-permeable membranes through solutions. The driving force for FO technology stems from water’s natural tendency to move between solutions characterized by different solute concentrations, since RO performs with external pressure alone. Water passes through the FO’s semi-permeable structure to transport NORMs [63].

FO membranes consist of two layers: an active layer controls water transport and slows solute movement, while a support layer supports membrane structure. These specialized membranes grant efficient NORM debris removal through their unique design structure while treating contaminated wastewater. FO shows effectiveness for NORM extraction because the system works with minimal hydraulic pressure, reducing operational costs and extending membrane lifespan [64].

Applications of FO in NORM Removal: Forward osmosis exhibits important potential to treat NORMs found in wastewater because it serves several industries, including mining, oil and gas, and nuclear power generation. The use of FO technology has demonstrated capability as a brine-concentration system for radioactive wastewater produced during hydraulic fracturing operations, where radium isotope removal reaches more than 90%. This dual benefit of volume reduction and high removal efficiency makes FO particularly suitable for high-salinity NORM-contaminated wastewater [65].

FO has proven effective for desalination operations that simultaneously eliminate NORM. Industrial wastewater treatment through FO, complemented by precipitation or adsorption, works as a dual solution for salinity management and radiological contamination reduction. An Australian pilot project implemented FO to process uranium mining wastewater, which exhibited successful uranium level reduction accomplishments measured against regulatory benchmarks [66]. Different examples demonstrate how FO maintains its flexibility to handle challenging wastewater situations. The treatment of industrial wastewater through FO technology has expanded because of new developments, which demonstrate its capability to handle oilfield wastewater while achieving complete water recovery [67,68]. FO technology, paired with membrane distillation systems, operates as an efficient water recovery system that maintains high energy performance [69,70].

Addressing these challenges requires the development of (1) draw solutions with lower regeneration energy requirements, such as thermolytic salts or magnetic nanoparticles, (2) membranes with reduced internal concentration polarization through optimized support layer structure, and (3) hybrid FO–membrane distillation systems that eliminate separate draw solution regeneration steps. Recent advances in thin-film composite membranes show promise for reducing these limitations.

## 6. Challenges to Widespread Adoption

Despite its promising potential, FO faces several obstacles to widespread adoption. The primary restriction to FO adoption remains the high expense associated with draw solution recovery because this step creates regenerable draw solutions that maintain process sustainability. FO technology alone cannot provide clean water since further processing through techniques like RO or thermal separation is required to isolate water from its draw solution, raising the operation expenses [71].

A major issue during membrane filtration processes is concentration polarization, which occurs when solute particles build up adjacent to the membrane surface, decreasing the osmotic force that drives filtration. The multifaceted internal CP, which arises inside the porous supporting framework of the membrane, presents more difficult challenges compared to the external CP. The efficiency of water transport diminishes, leading to a requirement for more regular maintenance through membrane cleaning and handling membrane structure alterations [72]. Scientists have focused their research on membrane material development through nanomaterial incorporation and surface property modification to reduce polarization effects and improve operational stability [73].

Fouling represents a critical problem in FO processes while handling wastewater that contains NORM. Membrane functionality reduces, and maintenance needs intensify when both organic material and radioactive particles deposit onto the surface. Radioactive fouling agents increase cleaning complexity through mandated regulatory compliance, together with specialized safety handling procedures [74].

## 7. Adsorption Techniques

The removal of NORMs from wastewater through adsorption techniques is a prevalent subject of research that employs activated carbon, biochar, and other modern adsorbents. The function of these materials rests on their ability to catch NORMs on their surface to lessen radioactive contaminant levels in water. High-surface-area activated carbon demonstrates remarkable adsorption ability because it possesses an extensive porous architecture capable of trapping radium, uranium, and thorium. Research shows that the ability to customize activated carbon’s hydrophobic and electrostatic features enables it to selectively bind specific radionuclides [75]. Research indicates that activated carbon removes uranium from water because of its metallic ion attraction capabilities.

The sustainable, organically produced carbon material known as biochar emerges as an alternative to traditional activated carbon. The surface functional groups on biochar, including carboxyl and hydroxyl, enable robust bonding with NORMs. Radium removal experiments indicate biochar created from agricultural leftovers achieves both eco-friendly treatment and low-cost application. Advanced materials, including zeolites and MOFs, together with functionalized nanoparticles, achieve high NORM adsorption capabilities because of their selective nature [76]. Comparative studies show that advanced materials achieve 10–20% higher removal efficiency than traditional adsorbents due to tailored surface chemistry and enhanced adsorption capacity [76,77].

Research findings show substantial advancements have occurred in radionuclide capture technologies through adsorbent material development. The physical and chemical processes of uranium adsorption in biochar-based materials lead to affordable, sustainable remediation solutions [78]. The selectivity of functionalized MOFs towards uranium (VI) and pertechnetate ions in radioactive waste solutions has been demonstrated by Lan et al. [79] and Patra et al. [80]. The surface properties of plasma-treated carbon materials have been enhanced to improve their ability to bind different types of radionuclides [81].

## 8. Factors Influencing Adsorption Effectiveness

The effectiveness of adsorption depends on several factors. Adsorptive capacity depends primarily on three material aspects, including pore size and surface area, as well as the presence of functional groups. Adsorbents that demonstrate both wide porosity and targeted functional groups provide superior performance in NORM adsorption. The bonding performance of adsorbents shifts when competing ions affect it alongside pH levels and salinity. Higher salinity conditions release adsorption rates through competitive ion interactions. Maximum adsorption efficiency depends on both the proper contact time between the rare earth metals and the adsorbent [82]. Recent studies on optimization have revealed the thermodynamic processes that control uranium adsorption behavior at different temperatures [83]. The research by Wang et al. [84] shows that heavy metal fixation and removal efficiency improve when the adsorbent and heavy metals stay in contact for longer periods. The stable bonds that form between metal ions and adsorbent functional groups during chemisorption result in better capture efficiency, according to Zhang et al. (2025) [85]. Table 3 presents the comparison between various technologies used for removal of NORM.

## 9. Precipitation and Sedimentation

Section 9 describes general precipitation and sedimentation processes, including natural settling and co-precipitation, while Section 10 focuses specifically on targeted chemical precipitation using reagents designed for selective radionuclide removal. Chemical precipitation (CP) is an effective method for separating NORMs from wastewater, relying on the addition of chemical agents that react with dissolved ions to form insoluble compounds. These compounds aggregate into larger particles, which can be removed through sedimentation or filtration. Several mechanisms, such as coprecipitation (CoP), Ostwald ripening, and pH alteration, are crucial for enhancing the efficiency of this process. CoP, for instance, occurs when a target compound like Sr^2+^ is removed by forming a precipitate with a reagent such as CaCO_3_, thus facilitating the removal process [92]. Wu et al. [93] demonstrated that introducing seed crystals, like CaCO_3_, significantly enhanced Sr^2+^ removal efficiency, achieving 99.88% removal after just one hour.

In addition to CoP, Ostwald ripening plays a key role in improving particle growth. In this mechanism, smaller particles dissolve and reattach to larger ones, thus increasing the size of the precipitate and improving its settling rate during sedimentation. This process facilitates easier aggregation of particles, which enhances the overall effectiveness of the precipitation. pH alteration also plays a significant role by promoting the formation of insoluble compounds like uranium hydroxide or radium carbonate, making it easier to separate these compounds from the liquid phase. To stabilize the separation process, membrane filtration (MF) is often integrated with CP. Wu et al. (2014) [93] found that using MF after precipitation ensured stable separation of Sr^2+^ and prevented the re-dissolution of precipitated ions [93].

Rogers et al. (2012) [94] further demonstrated the effectiveness of CP with MF for removing Cs from wastewater, using Fe_2_(SO_4_)_3_ as a coagulant, achieving a 92.7% removal efficiency. The study highlighted the importance of filter pore size, with smaller pores enhancing Cs removal post-precipitation. Additionally, Luo et al. [95] introduced a pellet co-precipitation micro-filtration (PCM) process that efficiently separated strontium from wastewater. In this process, strontium crystallized on CaCO_3_ seed particles, with Na_2_CO_3_ as a precipitating agent, and MF, enhanced by FeCl_3_, further improved the removal. This approach achieved a strontium decontamination factor (DF) of 577 and a concentration factor (CF) of 1958, demonstrating its potential for effective wastewater treatment [95].

Similarly, Zhao et al. [96] showed that flocculation sedimentation combined with microfiltration could remove more than 99.9% of plutonium from wastewater. By controlling the dosage of ferrous sulfate and adjusting the pH, a total α removal rate of 99.87% was achieved for mixed waste containing uranium, americium, and plutonium. UF, further removing colloids and suspended solids, was also found to enhance the overall treatment efficiency. This combination of methods highlights the effectiveness of chemical precipitation and filtration processes in removing NORMs from wastewater [96].

The current precipitation methods use hydroxyapatite composites and nanolayered metal oxides to enhance strontium and cesium removal efficiency through improved complexation, ion exchange, and precipitation processes [97,98,99]. The development of precipitation-based radioactive wastewater treatment methods becomes clear through these newly created technologies. Table 4 presents the results of studies involving precipitation-based radioactive wastewater treatment.

## 10. Chemical Precipitation

Chemical precipitation is a key method for removing radioactive contaminants from wastewater, particularly in the context of NORM-laden waste. This process involves adding chemical agents that react with dissolved radionuclides to form insoluble compounds. For example, in the hydrothermal reprocessing of liquid radioactive waste from nuclear power plants, chemical precipitation is utilized to separate radionuclides from the liquid phase [100]. Specific applications include the use of copper ferro(II)cyanide and sodium tetraphenylborate to remove radio cesium from environmental water samples [101]. Additionally, potassium ferrocyanide and ferrum nitrate are used in a two-stage precipitation technique to remove cesium ions through ion exchange and coagulation processes [102,103].

Osmanlioglu et al. [103] demonstrated that chemical precipitation is an effective method for removing radionuclides from wastewater, particularly Cs-137, Cs-134, and Co-60. In their two-stage process, chemical agents such as potassium ferrocyanide, nickel nitrate, and ferrum nitrate were used to achieve a decontamination factor of 66.5 for Cs-137, with 97.2% of the wastewater volume decontaminated. The method successfully reduced the volume of radioactive sludge, making it safer and more economical for disposal. This approach significantly lowered radiological risks, ensuring the treated wastewater could be discharged as clean water [103]. Oh et al. [104] investigated the removal of radionuclides such as 137Cs, 90Sr, and 60Co from complex acidic wastewater using potassium ferrocyanide and co-precipitation with BaSO4. Their results showed that the injection of Fe^3+^ significantly enhanced 137Cs removal, while effective hydroxide precipitation was used for 60Co. Additionally, 90Sr formed an isomorphous sulfate with Ba^2+^, requiring a large amount of BaSO_4_ for efficient removal, offering insights for reducing radioactive waste during nuclear decommissioning. The bulk removal of radionuclides through chemical precipitation works well, but researchers face two main obstacles in achieving better selectivity and reducing unwanted co-precipitation of non-target ions, especially for soluble radionuclides like cesium [105]. Scientists today work to enhance precipitating agent dosages and operational parameters for solving these problems.

## 11. Electrocoagulation

EC has gained attention for its potential in wastewater treatment, particularly in the removal of radionuclides like uranium. The process involves the use of metal anodes, such as iron, magnesium, or aluminum, which release metal ions into the solution. These ions react with water, forming metal hydroxides that can efficiently aggregate contaminants, including suspended particles and NORMs. One of the key advantages of EC is its ability to remove uranium by encapsulating it in ferric oxide/hydroxide colloids, which are formed during the process, thus improving removal efficiency [106].

Research by Li et al. [107] has demonstrated the enhanced uranium removal capability of EC when organic ligands with strong binding affinities are introduced into the system. These ligands facilitate the formation of stable complexes with uranium ions, specifically UO2^2+^, making it easier for the metal hydroxides to aggregate and remove uranium from wastewater. The role of chelating agents like ALR (alkali ligand reagents) has been further emphasized as crucial for improving uranium precipitation. While anodes such as iron (Fe) show high efficiency in capturing uranium through flocculation, magnesium (Mg) and aluminum (Al) anodes require the presence of ALR to mitigate challenges such as anode passivation and insufficient flocculation [107].

In addition to its role in removal, EC has proven effective for the recovery of uranium from low-concentration wastewater (<3.0 mg/L). Shao et al. [108] demonstrated that uranium could be successfully recovered from the precipitate using Na_2_CO_3_ + H_2_O_2_ eluents, showcasing EC’s dual functionality in both removing and recovering uranium. This makes EC a promising process for wastewater remediation, particularly in addressing contamination by uranium and other radionuclides in water systems.

Choi et al. [109] reinforced the efficiency of EC in removing suspended particles and uranium from wastewater, particularly when utilizing iron and aluminum anodes. In their case study, the iron–stainless steel anode system achieved a remarkably low uranium concentration of 5 μg/L, indicating the system’s effectiveness in reducing uranium levels. Furthermore, EC’s multi-objective optimization, which takes into account factors such as energy consumption and operational costs, makes it an environmentally sustainable and cost-effective solution. By meeting stringent regulatory limits for uranium in wastewater, EC proves to be a valuable tool in wastewater treatment and radioactive waste management. Recent developments in EC emphasize electrode material optimization and operational parameter adjustment to enhance uranium and radionuclide removal efficiency [110,111]. Integration of artificial intelligence for process optimization shows particular promise in improving EC performance through real-time data analysis and adaptive operational strategies [112].

The EC process, enhanced by the incorporation of chelating ligands like ALR, has shown great promise in removing and recovering uranium from wastewater. Its ability to effectively remove uranium while minimizing operational costs and energy consumption positions EC as an efficient and sustainable technology for addressing NORM contamination in water systems.

## 12. Advanced Membranes

Advanced membranes, including thin-film composite (TFC) and polymeric membranes, are engineered to efficiently remove NORM from liquid waste streams in nuclear, medical, and industrial applications. One of the most commonly utilized processes for NORM removal is RO, which employs TFC membranes known for their high selective permeability and rejection capabilities. These membranes are specifically designed to remove a wide range of radioactive ions, including beta and gamma emitters such as cesium (Cs), strontium (Sr), and cobalt (Co), as well as alpha emitters like actinides [113,114,115]. For example, in case studies, TFC RO membranes achieved up to 99% rejection of these hazardous elements, significantly reducing their concentration to levels below regulatory discharge limits, typically below 10 kBq/m^3^ for beta and gamma emitters and 1 kBq/m^3^ for alpha emitters [113].

Polymeric membranes, particularly those based on polyamide or polysulfone materials, are widely used in NF and UF, which focus on selective ion rejection. While NF membranes are designed to reject divalent and multivalent ions, UF membranes target the removal of larger macromolecules, colloids, and particles from radioactive waste. These polymeric membranes are particularly effective for decontaminating waters that contain complex mixtures of radioactive substances, as they offer a balance between permeability and selectivity, allowing only specific contaminants to be filtered out while maintaining high flow rates [116,117]. Table 5 presents a detailed evaluation of the main membrane technologies used for NORM extraction, which includes their rejection performance, operational parameters, and their tendency to clog, as well as their particular benefits and drawbacks for radioactive wastewater treatment. For instance, the Hydranautics CPA2-2540 membrane, a commonly used polymeric RO membrane, has shown excellent performance in decontaminating radioactive waste and efficiently removing a broad spectrum of radioactive ions [118].

**Figure 2:**
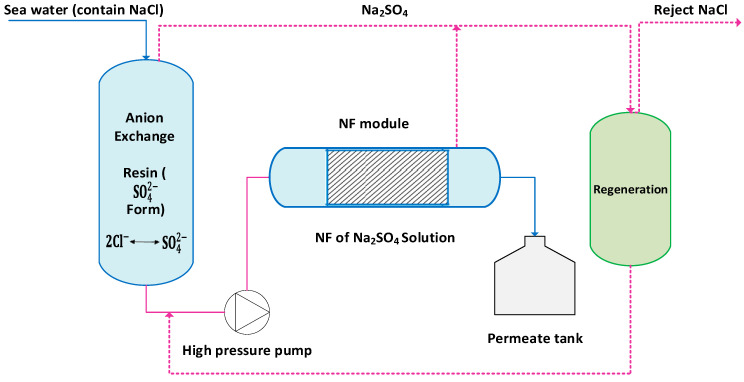
Conceptual schematic of hybrid ion-exchange nanofiltration (HIX-NF) desalination system illustrating chloride–sulfate exchange nanofiltration separation, and resin regeneration (adapted from [120]).

NF membranes are also used to remove hardness, total dissolved solids, turbidity, and bacteria. A critical review by Zhou et al. [119] studied different types of integrated systems of NF membranes and compared their cost in seawater desalination. Integrating NF systems with another desalination process was discussed. A hybrid system of NF and ion exchange, called HIX-NF, is shown in Figure 2. Such a system may reduce energy consumption by 50%.

**Table 5:** Comparison of membrane technologies for NORM removal through their performance parameters and operational requirements.

Membranes	Rejection	Operating Conditions	Fouling/NORM-Specific Issues	Radiation/Material Stability	Advantages for NORM Control	Limitations for NORM Control	Ref.
RO	High > 90–99% removal for dissolved Ra and U	High transmembrane pressure, sensitive to salinity and pH	Scaling and organic fouling, particulate-bound radionuclides can foul	Polymer embrittlement can cause long-term high radiation doses	An effective polishing process to achieve discharge limitations	Energy-intensive, produces concentrated brine with radioactive enrichment, and membranes need anti-fouling treatments	[14]
NF	Moderate to High (50–95%) based on ion charge/speciation (radium is frequently rejected due to its divalent nature)	Moderate pressure	Sensitive to organic and scale fouling, performance is sensitive to competing divalent ions (Ca and Mg)	Polymer concerns, Radiation effects are low at low doses	Lower energy. Selective rejection of divalent radionuclides. Minimize hardness and barium, which exists with Ra	Less consistent for monovalent ions	[121]
FO	Variable, high in feed-side retention of dissolved radionuclides when followed by adequate draw. Low performance for dissolved NORMs. Effective for colloid-bound radionuclides. recovery	Based on the draw selection and recovery method	Sensitive to organic and scale fouling	Membrane materials are comparable to thin-film composite; radiation stability considerations are similar	appropriate for high salinity produced water, lowered fouling, ideal as a first-stage to concentrate radionuclides for downstream treatment	Draw recovery may result in waste regeneration	[122]
Ultrafiltration (UF) Microfiltration (MF)	Low performance for dissolved NORMs. Effective for colloid-bound radionuclides	Low pressure	Cake formation, NORMs can easily foul the membrane	Radiation impact is negligible for low doses	Removes particulate-bound radioactivity and prevents downstream membrane fouling; low energy	Low efficiency in removing dissolved radionuclides	[75]

Additionally, novel membrane technologies such as membrane distillation (MD) and electrodeionization (EDI) have been explored for their potential in removing NORMs. MD, which utilizes porous hydrophobic membranes to separate volatile components from liquid waste, has shown promising results for treating radioactive isotopes like cobalt (Co), strontium (Sr), and cesium (Cs), with rejection factors exceeding 100,000 for these elements [123]. Similarly, EDI, which combines ion-exchange resins and electric fields to drive the ion removal process, has achieved up to 99.9% removal of specific radionuclides like cesium-137 (137Cs) from aqueous solutions. These advanced membrane technologies provide highly effective solutions for radioactive waste management, enabling nuclear plants, medical facilities, and research institutions to treat and dispose of radioactive materials in a controlled and environmentally safe manner [124,125,126,127]. The application of these membranes has been reported in various pilot-scale and plant-scale studies, which underscore their importance in managing radioactive contamination in liquid waste streams.

Advanced membranes, particularly amyloid-carbon hybrid filters, offer significant improvements over traditional radioactive wastewater treatment methods such as RO and NF. Conventional technologies, while capable of reducing the volume of wastewater, tend to concentrate contaminants in residual backwater, making the remaining water even more hazardous and difficult to manage. This poses a public health risk, especially in cases like the Fukushima disaster, where inefficient treatment led to the release of contaminated water into the Pacific Ocean [128]. In contrast, amyloid–carbon hybrid membranes, made from low-cost whey protein by-products, efficiently remove a wide range of radionuclides, including Tc-99m, I-131, and Lu-177, without generating secondary pollutants [129]. This adsorption-based filtering system offers an effective and sustainable solution, with exceptional performance in actual wastewater tests conducted at Inselspital, Switzerland. Moreover, these membranes are highly reusable, scalable, and cost-effective, making them a promising technology for large-scale radioactive wastewater treatment in hospitals and nuclear facilities [128].

Recent innovations in membrane distillation demonstrate significant potential for treating low-level radioactive liquid waste, with vacuum membrane distillation systems achieving effective separation of radionuclides under mild operational conditions [130]. Hybrid membrane designs incorporating nanofibers and enhanced hydrophobic properties further improve separation efficiency and durability for radioactive waste applications [131], while optimization of operational parameters such as temperature and flow rates continues to enhance transmembrane mass transfer performance [132].

## 13. Evaluating Efficiency and Considerations

Efficiency indicators such as removal efficiency, throughput, and operational costs play a significant role in evaluating the performance and sustainability of various technologies used for adsorbing radionuclides. Removal efficiency, often quantified as the percentage of contaminants removed, directly impacts the effectiveness of an adsorbent. For instance, Khandaker et al. [133] found that air-oxidized bamboo charcoal demonstrated a removal efficiency of 98% for cesium, highlighting the high effectiveness of this adsorbent in specific conditions (pH 2–12 and temperatures of 288–308 K). Similarly, Teng et al. [134] achieved a removal efficiency of 97.6% for uranium using modified rice stems under optimal conditions (pH 4 and 298 K). These studies underline that the removal efficiency can vary depending on adsorbent type, pH level, temperature, and contaminant concentration, making it essential to tailor conditions for maximum performance.

Throughput, the volume of wastewater processed within a specific timeframe, is another critical efficiency indicator. Technologies like activated carbon and CNTs often have higher throughput capabilities compared to newer adsorbents such as MOFs, which are still in the research phase. However, their economic feasibility is not yet well-established, and their scalability remains a concern. For example, Yavari et al. [135] demonstrated the use of oxidized multiwall CNTs for strontium ion sorption, achieving a relatively high adsorption capacity (6.62 mg/g), but at a lower removal efficiency (85%) compared to bamboo charcoal.

Operational costs are integral to determining the long-term viability of adsorbents. Activated carbon is recognized for its cost-effectiveness, especially when derived from low-cost materials like rice husks [136]. In contrast, adsorbents like MOFs and CNTs are still associated with higher production costs due to their complex synthesis methods. When analyzing the economic viability, life cycle assessments (LCAs) are critical for determining the overall environmental impact and costs over time. For instance, while MOFs may offer superior adsorption capabilities, their high manufacturing costs and limited scalability may hinder their commercial application, particularly in large-scale operations. Therefore, it is essential to evaluate the balance between removal efficiency, throughput, and operational costs for a more sustainable approach to radionuclide remediation.

The current research focuses on developing adsorbent systems that provide efficient mass transfer and adsorption performance at affordable costs [97,137]. Research into new adsorbent technologies shows that industrial materials need to prove both effective removal performance and operational suitability for industrial applications of NORMs [32].

## 14. Challenges and Comparative Studies

Implementing advanced membrane technologies for the removal of NORM in wastewater faces several significant challenges. One of the primary issues is membrane fouling, which occurs when contaminants accumulate on the membrane surface, reducing its effectiveness. This is especially problematic in radioactive wastewater, as the high concentrations of dissolved solids and complex colloidal matter can lead to rapid fouling. As a result, frequent cleaning and potential replacement of membranes are required, increasing operational costs and downtime. Additionally, achieving high rejection rates for specific radioactive species while maintaining an efficient flow rate is difficult. Membranes must be tailored to selectively reject harmful ions, such as cesium and strontium, while allowing non-radioactive ions to pass through. This requires precise engineering to ensure the membrane material is sufficiently selective [29,30].

Experimental studies quantify these challenges. Fouling significantly reduces membrane flux within days when treating untreated NORM wastewater, though pre-treatment substantially mitigates this decline. Radiation exposure progressively degrades membrane materials, particularly polymeric membranes, reducing operational lifetime in high-activity streams. Scaling becomes problematic when calcium and barium concentrations are elevated, forming sparingly soluble sulfate precipitates that reduce flux. Energy requirements increase substantially with feed water salinity, making high-TDS streams (>50,000 mg/L) particularly energy-intensive to treat with pressure-driven membranes.

Another challenge lies in the material properties of the membranes. Thin-film composite (TFC) and polymeric membranes, commonly used in RO and NF systems, must withstand the radiation-induced degradation and chemical attacks from the wastewater, especially in acidic or alkaline environments [72]. Radiation can weaken the membrane structure over time, leading to a reduction in its lifespan. Furthermore, scaling issues often occur as salt and radioactive mineral deposits on the membrane surface, compromising efficiency. High operational pressures are also required for RO and NF processes, contributing to energy inefficiency and the need for advanced, energy-efficient designs. Finally, managing the concentrated radioactive waste generated during the filtration process adds an additional complexity, requiring safe disposal methods to prevent environmental contamination. These challenges underscore the need for continued research and innovation to improve the performance and sustainability of membrane systems for NORM removal [52].

The research into fouling prevention techniques has introduced two new methods, which use dynamic membrane layers as a pre-deposition barrier and chemical treatments to modify water characteristics for membrane protection [138,139]. Research shows that inorganic and geopolymeric membranes engineered for radiation resistance are ready for nuclear waste management because they stay structurally sound when exposed to ionizing radiation [140].

## 15. Critical Technology Comparison, Future Directions and Recommendations

Direct comparison of NORM removal technologies is complicated by inconsistent reporting of experimental conditions across studies. While Table 1 normalizes available performance data, several factors affect technology selection for industrial applications.

Laboratory studies typically report higher removal efficiencies than industrial systems due to idealized conditions, controlled parameters, and the absence of long-term fouling. Salinity significantly impacts performance: membrane technologies show substantial efficiency decline when TDS exceeds 50,000 mg/L, yet many studies use lower-salinity feed water that poorly represents oil- and gas-produced water.

Based on synthesized data, we recommend ion exchange or RO for low-salinity wastewater (TDS < 15,000 mg/L), NF or hybrid systems for medium salinity (15,000–35,000 mg/L), and FO or adsorption for high-salinity applications (>35,000 mg/L), though industrial validation remains limited. Hybrid systems offer higher removal efficiency but incur higher capital costs, though lower operating costs may offset this over extended operation.

### 15.1. Technology Selection Framework

Selecting appropriate NORM treatment technologies requires a systematic approach. First, wastewater characteristics must be thoroughly assessed, including TDS, pH, radionuclide concentrations, competing ions, and organic content. Based on salinity levels, practitioners can identify suitable primary technologies as outlined above. However, if single-technology systems cannot meet regulatory discharge limits, or if high organic content (TOC > 20 mg/L) poses significant fouling risks, hybrid configurations should be considered. Finally, the economic analysis must account for the total cost of ownership, including waste disposal expenses, rather than focusing solely on capital investment. This framework enables matching treatment strategies to specific wastewater characteristics and regulatory requirements.

### 15.2. Critical Research Gaps and Future Directions

Critical gaps persist: Long-term performance data are scarce, radioactive waste disposal costs are rarely quantified, and systematic optimization of hybrid configurations requires further investigation.

There is a pressing need for research focused on developing novel materials to enhance the removal of NORM from wastewater. Promising avenues include the design of radiation-resistant, high-performance membranes capable of enduring harsh operational conditions without significant degradation. Materials such as graphene-based membranes, known for their superior permeability and selectivity, and polymer blends that exhibit enhanced resistance to radiation and fouling, may offer significant improvements in membrane longevity and efficacy. Additionally, hybrid treatment approaches, integrating membrane filtration with complementary technologies such as EC or adsorption, could further optimize NORM removal, offering a more comprehensive and cost-effective solution. Innovations in membrane surface modifications, exceptionally functional coatings that selectively capture radioactive particles, warrant further exploration to enhance treatment performance.

From a regulatory perspective, the establishment of standardized protocols for NORM treatment processes is essential to ensure both safety and efficacy. Regulatory frameworks should address the treatment of NORM-laden wastewater, with particular attention to the safe disposal of concentrated radioactive waste. Furthermore, the implementation of policies that incentivize the development and commercialization of advanced NORM removal technologies—such as research grants, tax incentives for sustainable technologies, and funding for pilot-scale projects—could accelerate their broader adoption. Collaborative efforts between industrial sectors and research institutions may facilitate translating innovative solutions into practice, promoting the widespread deployment of effective, scalable NORM treatment technologies.

The future of NORM wastewater treatment will benefit from sustainable advanced solutions combining digital optimization with novel materials and hybrid systems. Machine learning approaches can optimize treatment performance and operational efficiency [141]. Advanced membrane systems integrated with oxidation processes show promise for challenging radionuclide removal applications [142], while biochar-based methods offer sustainable alternatives for metal extraction, including radionuclides [143]. Photocatalytic approaches, when combined with membrane technologies, demonstrate potential for enhanced NORM treatment [144]. These emerging technologies address critical challenges in industrial wastewater management while advancing towards sustainable solutions.

## 16. Conclusions

The sustainable management of NORMs in industrial wastewater remains a pressing challenge for both environmental protection and public health. This review highlights that no single treatment approach can achieve complete radionuclide removal under varying operational and wastewater conditions. Instead, hybrid configurations that combine membrane filtration, adsorption, EC, and precipitation consistently demonstrate higher removal efficiencies, achieving 85–97% removal for uranium and 78–97% removal for radium depending on technology and operating conditions, with hybrid systems showing 15–25% improvement over single-technology approaches. Nanostructured membranes and FO stand out as particularly promising due to their high selectivity, low energy demand, and capability to treat saline and complex waste streams. However, the implementation of these systems depends on resolving multiple technical and economic obstacles.

The technologies reviewed here are at different stages of commercial readiness. RO, NF, ion exchange, and activated carbon are well-established and available for immediate industrial implementation. FO and EC show promise at pilot scale but need further refinement for widespread NORM treatment. Advanced nanomaterials such as MOFs, CNTs, and graphene-based membranes remain largely confined to laboratory research, with practical deployment hindered by high costs, complex regeneration requirements, and unresolved long-term stability issues.

Despite these advances, operational barriers such as membrane fouling, draw-solution recovery in FO systems, secondary waste generation, and the absence of standardized disposal protocols for radioactive waste continue to limit large-scale adoption. Future research should emphasize the development of radiation-resistant, recyclable membrane materials and cost-effective sorbents such as biochar and metal–organic frameworks that can function at industrial levels while remaining economically viable. Moreover, integrating digital process control and artificial intelligence can improve efficiency, predictive maintenance, and reduce the overall environmental impact.

A coordinated framework linking research institutions, industry, and regulators is essential to translate laboratory advances into scalable, field-ready technologies. Such collaboration will enable the safe, economically viable, and environmentally responsible management of radioactive wastewater in alignment with global sustainability objectives, particularly UN Sustainable Development Goal 6 on clean water and sanitation.

## Figures and Tables

**Table 1:** Comparative performance of NORM removal technologies.

Technology	U Removal (%)	Ra Removal (%)	Optimal pH	Energy (kWh/m^3^)	TDS Limit (mg/L)	Reference
EC	85–92	78–85	6–8	2.5–4.0	<15,000	[20]
Ion Exchange	88–95	82–90	4–6	0.8–1.5	<10,000	[22]
RO	94–97	94–97	7–8	4.0–6.0	<35,000	[35]
NF	88–94	88–94	7–8	2.0–3.0	<35,000	[36]
Biochar Adsorption	75–88	70–82	5–7	0.5–1.0	<20,000	[37]
MOFs	90–96	88–94	5–7	1.0–2.0	<25,000	[39]

**Table 2:** Quantitative advantages and limitations of NORM removal technologies.

Technology	Removal Efficiency (%)	Energy (kWh/m^3^)	TDS Limit (mg/L)	Primary Limitation
RO	94–97	4–6	<50,000	High energy, brine disposal
NF	88–94	2–3	<35,000	Performance drops at high TDS
Ion Exchange	88–95	0.8–1.5	<10,000	Competing ions reduce efficiency
EC	85–92	2.5–4	<15,000	Electrode corrosion
Biochar	75–88	0.5–1	<20,000	Capacity decline at high salinity
FO	87–92	3–5	>30,000	Draw solution regeneration

**Table 3:** Different technologies in the removal of NORM: advantages and disadvantages.

Technologies for Removal of NORM	Pros	Cons	Economic & Environmental Considerations	Examples	Removal Efficiency
Nanotechnology Membranes [35]	High efficiency in NORM removal; low energy consumption; resistant to fouling; scalable.	High initial costs; potential material degradation under extreme conditions.	Reduces operational energy use; requires investment in advanced nanomaterials and sustainable disposal of used membranes.	Graphene oxide: Pilot in India, CNTs: Case study in the US, MOFs: Pilot in China, TiO_2_: Municipal applications in Europe.	U: 95–97%, Ra: 92–95%
FO [86]	Low energy requirements; effective for high-salinity wastewater; minimizes fouling.	Concentration polarization limits performance and results in high costs for specialized membranes.	Energy-efficient compared to RO; brine management can have environmental challenges.	Literature highlights FO’s potential in desalination and brine concentration applications.	U: 88–92%, Ra: 87–92%
Adsorption [87]	Cost-effective for low-concentration pollutants; sustainable with biochar or activated carbon.	Limited to specific contaminants; regeneration challenges; reduced effectiveness in complex matrices.	Affordable for large-scale use with biochar; limited chemical waste generation, but may require frequent replenishment.	Studies on biochar and advanced adsorption materials in water treatment.	U: 75–88%, Ra: 70–82%
Precipitation & Sedimentation [88]	Simple, low-cost technology; suitable for bulk contaminant removal.	Inefficient for low-concentration NORM; requires post-treatment to meet stringent standards.	Economical but generates significant sludge; disposal requires environmental safeguards.	Commonly used in industrial wastewater treatment, particularly in initial treatment phases.	U: 70–85%, Ra: 65–80%
Chemical Precipitation [66]	High efficiency in removing specific radionuclides like radium and uranium.	Requires precise dosing; generates hazardous sludge; higher operational costs.	Sludge management is costly and involves chemical input and potential secondary contamination.	Applied in uranium mining wastewater treatment and other industrial contexts.	U: 80–90%, Ra: 75–88%
EC [89]	Efficient for removing metal ions and NORMs; minimal chemical use; scalable.	High energy demand; electrode corrosion and maintenance can increase costs.	Relatively sustainable if renewable energy is used; avoids excessive chemical use, reducing the environmental footprint.	Documented in pilot-scale and municipal-scale operations.	U: 85–92%, Ra: 78–85%
Advanced Membranes [90]	High selectivity and efficiency; customizable for specific pollutants; scalable.	Expensive materials; fouling issues are still a concern despite advancements.	Investment in R&D and production costs is high; long-term operational efficiency can offset initial costs.	Nanotech membranes, MOFs, CNTs, and TiO_2_ examples are mentioned above.	U: 90–96%, Ra: 88–94%
Nanotech Membranes [91]	High contaminant removal rates; improved operational sustainability; minimal fouling.	Requires a skilled workforce for operation and maintenance; disposal of nanomaterials remains challenging.	Advanced technologies like GO, CNT, and TiO_2_ balance efficiency with environmental considerations if implemented effectively.	Pilot projects and studies showcasing diverse applications of nanotech membranes in industrial and municipal settings globally.	U: 95–97%, Ra: 92–95%

**Table 4:** Removal efficiency of radioisotopes from different water samples using precipitation and filtration methods.

Mechanism	Precipitating Agent/Crystal Seed	Targeting Compounds (ICon)	Removal Efficiency (%)	Sample Type & Other Properties	References
CoP with MF	Na_2_CO_3_ (1000 mg/L)/CaCO_3_ (0.34 g/L)	Sr^2+^ (5 mg/L)	99.88% after 1 h	Raw water, temperature 23–26 °C, surface area = 0.5 m^2^, membrane pore diameter = 0.22 μm	[93]
Dilution–Precipitation	Sodium tetraphenylborate (NaTPB)/Fe_2_(SO_4_)_3_ as coagulant	^133^Cs/^137^Cs (100 mg/L)	92.7% after 2 h	Wastewater, 100 rpm, pH = 5.5	[94]

## Data Availability

The authors confirm that the data supporting the findings of this study are available within the article.
